# Dual-depth 25-pin insulated microneedle radiofrequency for moderate-to-severe periorbital wrinkles

**DOI:** 10.1007/s10103-026-04942-0

**Published:** 2026-07-17

**Authors:** Kyung Tae Bae, So Yoon Park, Yerin Park, Kyu-Ho Yi

**Affiliations:** 1 It’s Me Clinic, Sejong, Korea, Republic of; 2 Cheongdam Eclatde Clinic, Seoul, Korea, Republic of; 3 Medical Research, Wonju, Korea, Republic of; 4 You and I Clinic, Seoul, Korea, Republic of; 5 Division of Anatomy & Developmental Biology, Department of Oral Biology, Yonsei University College of Dentistry, Seoul, Korea, Republic of

**Keywords:** Microneedle radiofrequency, Periorbital rejuvenation, Dual-depth RF delivery, Skin elasticity, Fitzpatrick Wrinkle Assessment Scale

## Abstract

To evaluate efficacy and safety of dual-depth 25-pin insulated microneedle radiofrequency (RF) treatment for periorbital wrinkles and skin elasticity. This observational study included 32 adults aged 30–60 years with baseline Fitzpatrick Wrinkle Assessment Scale (FWAS) scores of 4 or higher. Patients underwent three microneedle RF treatment sessions at 4-week intervals. The protocol employed a duty-controlled, dual-shot mechanism to sequentially deliver thermal energy to 1.5 mm and 1.0 mm depths within a single needle insertion. Outcomes were assessed at baseline and weeks 16 and 24 using blinded FWAS grading, Antera 3D imaging, Cutometer R2/R5/R7, patient satisfaction, and safety monitoring. The mean FWAS score decreased from 5.44 ± 0.80 at baseline to 3.38 ± 0.61 at week 24 (*p* < 0.001). Quantitative imaging revealed relevant reductions in average wrinkle depth, maximum wrinkle depth, and the indentation index, while R2, R5, and R7 increased throughout follow-up (all *p* < 0.001). At week 24, 90.63% of patients were satisfied or very satisfied. The mean procedure time was 7.2 ± 1.4 min, and the mean pain score was 3.3 ± 0.9. Adverse events were limited to transient mild erythema and edema, resulting in an average downtime of 1.5 ± 0.5 days. The dual-depth, 25-pin insulated microneedle RF protocol demonstrated clinical utility and a favorable safety profile as a minimally invasive option for periorbital rejuvenation. By delivering targeted dermal thermal stimulation without repeated mechanical passes, the procedure improved wrinkle severity and skin elasticity with short treatment time and limited recovery burden.

## Introduction

Periorbital wrinkles are early indicators of facial aging that contribute significantly to a fatigued appearance. Their etiology is multifactorial, involving intrinsically thin eyelid skin, repetitive orbicularis oculi muscle contractions, and photoaging-induced dermal degradation [4, 9, 11, 17]. Because the periorbital region typically presents a complex mixture of dynamic rhytides and static creases, therapeutic approaches targeting a single pathophysiological mechanism often yield insufficient correction.

Conventional rejuvenation strategies are dictated by the predominant clinical presentation. Surgical interventions, such as blepharoplasty, are reserved for marked skin redundancy or eyelid ptosis [9]. Conversely, while botulinum toxin effectively targets dynamic wrinkles through chemodenervation, it does not induce structural dermal remodeling [8]. Energy-based surface treatments can improve skin texture but require cautious application in the delicate periorbital area due to risks of prolonged erythema and hyperpigmentation [2, 12]. Therefore, a minimally invasive modality capable of inducing dermal extracellular matrix remodeling while sparing the epidermis remains a critical need for patients seeking substantial improvement with limited downtime.

Microneedle radiofrequency (RF) offers a targeted approach to address this therapeutic gap. By creating physical micro-channels and delivering controlled thermal energy directly into the dermis, microneedle RF stimulates a wound-healing cascade that promotes neocollagenesis and neoelastogenesis [5, 11, 14]. However, achieving consistent energy delivery in the periorbital region is challenging due to its highly contoured and mobile topography. To overcome these anatomical barriers, the RF protocol evaluated in this study incorporates vacuum-assisted fixation to ensure stable tissue contact, alongside a double-shot emission mechanism. This dual-depth delivery strategy is designed to simultaneously stimulate both superficial and deep dermal layers within a single insertion cycle, thereby maximizing thermal remodeling while minimizing repetitive mechanical trauma.

Accordingly, this study evaluated the clinical efficacy and safety of a 25-pin insulated microneedle RF protocol in adults with moderate-to-severe periorbital wrinkles, defined as an FWAS score of 4 or higher. After three treatment sessions at 4-week intervals, outcomes were assessed at weeks 16 and 24 using clinical wrinkle grading, 3D imaging, skin elasticity measurements, patient satisfaction, and safety monitoring.

## Method

### Study design and participants

This study was designed as a retrospective analysis of prospectively collected clinical data to evaluate the outcomes and safety of a microneedle radiofrequency (RF) procedure for periorbital rejuvenation. Clinical records of patients who underwent three consecutive RF treatment sessions at 4-week intervals, with standardized follow-up assessments at 16 and 24 weeks post-initial treatment, were analyzed.

A total of 32 patients initially underwent treatment. All patients completed three treatment sessions; however, all 32 patients were included in the final analysis because they also completed both follow-up evaluations. No dropouts occurred during the study period. The study was approved by the Institutional Review Board (Approval No. P01-202511-01-072) and conducted in accordance with the Declaration of Helsinki. Written informed consent was obtained from all patients prior to the procedure.

The study population comprised 28 females and 4 males, with a mean age of 45.8 ± 7.2 years. All evaluated patients exhibited Fitzpatrick skin phototypes ranging from II to IV.

All evaluated patients exhibited Fitzpatrick skin phototypes ranging from II to IV.

## Microneedle RF system

For the improvement of periorbital wrinkles and skin elasticity, a microneedle RF system (ELLISYS SENSE; CHUNGWOO Co., Ltd., Seoul, Korea) was utilized. This system operates in bipolar mode (2 MHz) for all treatments in this study. The system features an RF frequency of 1–2 MHz and a maximum power output of 70 W. Adjustable operating parameters include shot time (0.05–1800 ms), needle depth (0.5–8.2 mm), interval time (0.3–3.0 s), and vacuum levels.

Various needle tips can be employed depending on the treatment objective and anatomical site, including 25-pin insulated, 49-pin non-insulated, 1-pin monopolar, and 36-pin insulated needles. The selection of the tip is determined by the target surface area, desired depth, and energy delivery mode. Given the thin skin and restricted surface area of the eyelids and periorbital region, the 25-pin insulated needle tip was selected for this study. This specific configuration emits RF energy predominantly from the distal ends of the needles, making it optimal for delivering selective thermal stimulation at superficial target depths.

The 25-pin insulated microneedle cartridge used in this study has an outer needle diameter of approximately as provided by manufacturer, an effective penetration length range of 0.5–8.2 mm, and an insulated shaft with exposed distal tip designed to concentrate RF energy at the dermal target layer. The tip geometry is characterized by a fine tapered configuration to facilitate controlled dermal penetration with minimal epidermal trauma. These specifications were consistent with the manufacturer’s technical documentation for the Ellisys Sense system.

During the procedure, vacuum-assisted fixation was utilized to ensure close adherence of the tip to the skin surface prior to needle insertion. RF energy was delivered via a duty-controlled, dual-shot mechanism. This technique sequentially delivers a primary and a secondary RF impulse to distinct tissue depths within a single needle insertion cycle. Duty control, which segments a single RF emission into shorter sub-shots, was employed to mitigate excessive localized heat accumulation associated with continuous RF irradiation.

The principal parameters of this system encompass RF level, emission time, duty cycle, needle depth, and interval time. The RF level signifies the intensity of the power output, while the emission time denotes the duration of RF energy delivery. The duty cycle represents the ratio of active RF emission within the designated emission time. Needle depth defines the penetration extent of the microneedles, and the interval time is the inter-pulse delay between consecutive shots, which is a critical factor in regulating thermal accumulation within the tissue.

## Procedures

Patients underwent a total of three microneedle RF treatment sessions spaced at 4-week intervals. The initial treatment was administered at the baseline visit, followed by subsequent sessions at week 4 and week 8. Follow-up evaluations were conducted at week 16 and week 24.

The treatment zone encompassed the entire periorbital area, including the upper eyelid, lower eyelid, infraorbital region, and lateral canthus. Prior to each procedure, the treatment area was cleansed and disinfected with 70% isopropyl alcohol. A topical anesthetic cream (EMLA) was then applied and maintained for 30 min. Immediately before the procedure, residual anesthetic was meticulously removed, and the area was re-disinfected.

**Fig. 1:**
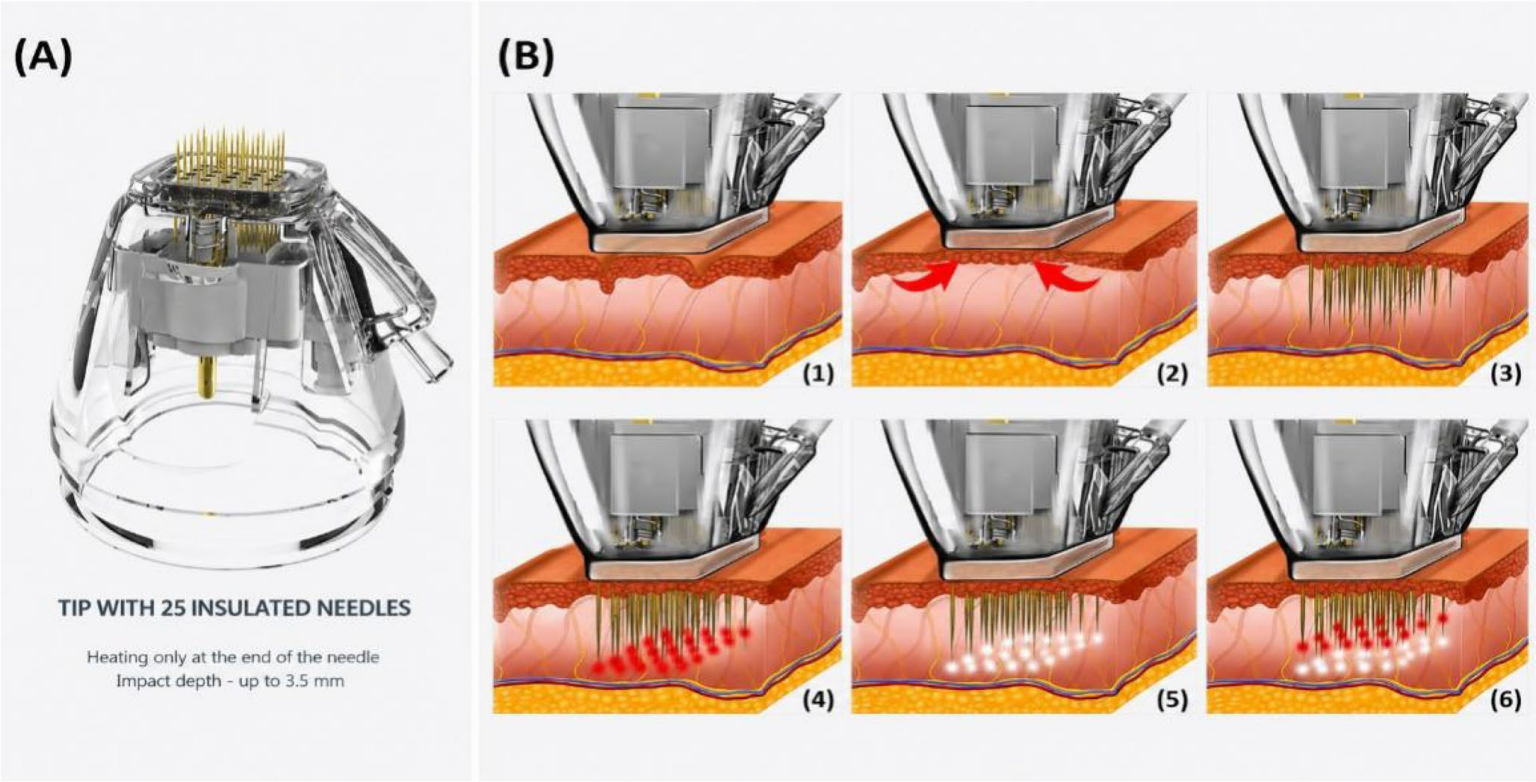
Schematic illustration of the 25-pin needle tip and delivery process of double-shot microneedle radiofrequency treatment. (**A**) A 25-pin insulated needle tip. The insulated needle configuration is designed to deliver RF energy predominantly at the distal needle tip, allowing selective thermal stimulation at the preset tissue depth. (**B**) Sequential process of vacuum-assisted microneedle RF delivery. [1] The tip is placed in contact with the skin surface, [2] the target tissue is suctioned and stabilized, [3] the microneedles penetrate to the preset depth, [4] the first RF impulse is delivered at a deeper tissue level, creating deep coagulation points, [5] the needles are repositioned to a more superficial depth, and [6] the second RF impulse is delivered at the superficial level, allowing sequential RF delivery at two different depths within a single needle insertion cycle

The procedure was performed with the patient in a supine position. Sterile ocular protection was applied using metal corneal shields in all patients prior to treatment of the upper and lower eyelid regions to prevent accidental ocular injury. The operator gently positioned the needle tip flush against the skin surface, and activated the suction mechanism to stabilize the tissue. Subsequent to stabilization, needle insertion and RF emission were executed. The treatment was administered utilizing a spot-by-spot stamping technique, wherein the tip was lifted and relocated to the adjacent area following each shot. The sequence progressed along the upper eyelid, lower eyelid, lateral canthus, and infraorbital region, maintaining an overlap of approximately 50% between adjacent shots. Utilizing the “Double Shot” function, sequential primary and secondary RF impulses were delivered to different depths during a single insertion cycle (Fig. 1). Specific treatment parameters are detailed in Table 1.

**Table 1:** Treatment Parameters for Microneedle RF

Setting	1 st impulse	2nd impulse
Needle tip	Insulated 25-pin	Insulated 25-pin
RF frequency	2 MHz	2 MHz
RF level	5	4
Emission time	200 ms	150 ms
Duty cycle	80%	80%
Needle depth	1.5 mm	1.0 mm
Interval time	0.3 s	0.3 s
Suction	On	On
Overlap	Approximately 50%	Approximately 50%

After treatment, a soothing cream was applied, and patients were instructed to avoid rubbing, eye makeup, exfoliants, retinoids, acidic skincare products, heat exposure, vigorous exercise, and alcohol for 24–48 h. Patients were advised to report any unexpected post-treatment reactions.

## Outcome assessment

### Clinical wrinkle assessment

The clinical severity of periorbital wrinkles was evaluated using the Fitzpatrick Wrinkle Assessment Scale (FWAS), a clinically validated scale for standardized wrinkle severity assessment [15]. The assessment encompassed the entire periorbital region, including the upper and lower eyelids, infraorbital area, and lateral canthus. Standardized photographs (frontal, bilateral oblique, and close-up periorbital views) were obtained. All photographs were acquired under standardized clinical conditions using consistent camera settings, fixed lighting environment, and controlled patient positioning to minimize variability in image acquisition. Two independent, blinded evaluators, uninvolved in the procedures and unaware of the treatment timeline, assessed the wrinkle severity. The mean value of the left and right measurements was utilized as the representative subject-level score.

### Three-dimensional imaging analysis

Quantitative analysis of periorbital wrinkles was conducted using the Antera 3D^®^ CS system (Miravex Limited, Dublin, Ireland), which enables objective measurement of wrinkle depth and skin surface characteristics [13]. Imaging was performed separately for the left and right sides, capturing one entire periorbital area per image. The primary analytical parameters were average wrinkle depth, maximum wrinkle depth, and the indentation index. The values derived from both sides were averaged to yield a single representative value per patient.

## Skin elasticity assessment

Skin elasticity was measured utilizing the Cutometer^®^ Dual MPA 580 (Courage + Khazaka electronic GmbH, Cologne, Germany). Measurements were acquired bilaterally on predefined zones: the upper eyelid, lower eyelid/infraorbital area, and lateral canthus. Three consecutive measurements were obtained at each anatomical site, and the mean values were calculated. The primary parameters analyzed were R2, R5, and R7, which reflect skin elasticity and resilience. Bilateral values were averaged for subject-level analysis. Measurement conditions for Cutometer included a 2 mm probe diameter, standard negative pressure of 450 mbar, and an on/off time of 2.0/2.0 s for each measurement cycle.The overall periorbital elasticity score was derived from the mean of the upper eyelid, lower eyelid/infraorbital, and lateral canthus measurements.

## Patient satisfaction

Patient satisfaction was assessed at the follow-up visits using a 5-point Likert scale. The evaluation encompassed satisfaction with wrinkle reduction, elasticity improvement, overall enhancement of the periorbital appearance, and general satisfaction with the procedural outcomes. Scores were defined as 1 (very dissatisfied), 2 (dissatisfied), 3 (neutral), 4 (satisfied), and 5 (very satisfied).

### Pain and safety assessment

Intraprocedural pain was evaluated immediately post-treatment using a 10-point visual analogue scale (VAS), with 0 indicating no pain and 10 representing the maximum tolerable pain. Safety assessments were performed immediately post-procedure and at all follow-up visits. Monitored variables included erythema, edema, petechiae, bruising, crusting, burns, hyperpigmentation, sensory alterations, signs of infection, and any other adverse events. The occurrence, severity, onset time, duration, necessity for medical intervention, and resolution status of each adverse event were documented.

### Statistical analysis

All statistical analyses were performed using SPSS version 26.0. Continuous variables were assessed for normality using the Shapiro–Wilk test and presented as mean ± SD or median with interquartile range, as appropriate. Categorical variables were summarized as frequencies and percentages. Longitudinal changes in FWAS, Antera 3D parameters, and Cutometer values across baseline, week 16, and week 24 were analyzed using repeated-measures ANOVA or the Friedman test, according to data distribution. When significant temporal differences were identified, Bonferroni-corrected post-hoc comparisons were performed. Inter-rater reliability for FWAS scoring was assessed using the intraclass correlation coefficient or weighted kappa. Pain, patient satisfaction, and adverse events were summarized descriptively. A two-sided p-value < 0.05 was considered statistically significant.

## Results

### Patient demographics

The datasets of 32 patients who completed all three microneedle RF treatment sessions and the scheduled follow-up evaluations at week 16 and week 24 were included in the final analysis. Baseline demographic characteristics are summarized in Table 2.

**Table 2:** Patient Demographics and Baseline Characteristics

Variable	Value
Total patients, n	32
Female, n (%)	28 (87.50%)
Male, n (%)	4 (12.50%)
Age, years, mean ± SD	45.80 ± 7.20
Fitzpatrick skin phototype II, n (%)	6 (18.75%)
Fitzpatrick skin phototype III, n (%)	17 (53.13%)
Fitzpatrick skin phototype IV, n (%)	9 (28.12%)
Baseline FWAS score, mean ± SD	5.44 ± 0.80

### Clinical wrinkle severity

Periorbital wrinkle severity was assessed using the FWAS at baseline, week 16, and week 24. The mean FWAS score was 5.44 ± 0.80 at baseline, 3.81 ± 0.74 at week 16, and 3.38 ± 0.61 at week 24. Longitudinal analysis demonstrated a temporal change in FWAS scores across the three time points, with the global test result summarized in Table 3.

**Table 3:** Longitudinal Changes in Clinical and Quantitative Outcome Measures

Outcome variable	Baseline	Week 16	Week 24	*p*-value
FWAS score	5.44 ± 0.80	3.81 ± 0.74	3.38 ± 0.61	< 0.001
Average wrinkle depth (mm)	0.22 ± 0.05	0.16 ± 0.04	0.14 ± 0.03	< 0.001
Maximum wrinkle depth (mm)	0.65 ± 0.12	0.48 ± 0.09	0.42 ± 0.08	< 0.001
Indentation index	29.85 ± 4.50	21.40 ± 3.80	19.15 ± 3.20	< 0.001
R2	0.55 ± 0.08	0.66 ± 0.06	0.72 ± 0.05	< 0.001
R5	0.35 ± 0.06	0.44 ± 0.05	0.49 ± 0.04	< 0.001
R7	0.31 ± 0.05	0.38 ± 0.04	0.43 ± 0.04	< 0.001

*Values are presented as mean ± SD. RM-ANOVA*,* repeated measures analysis of variance; FWAS*,* Fitzpatrick Wrinkle Assessment Scale.*

**Fig. 2:**
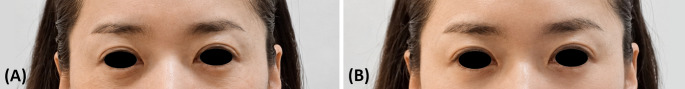
Clinical photographs of a 37-year-old female patient with Fitzpatrick skin type III. (**A**) Baseline (FWAS: 5). (**B**) Week 24 (FWAS: 3)

**Fig. 3:**
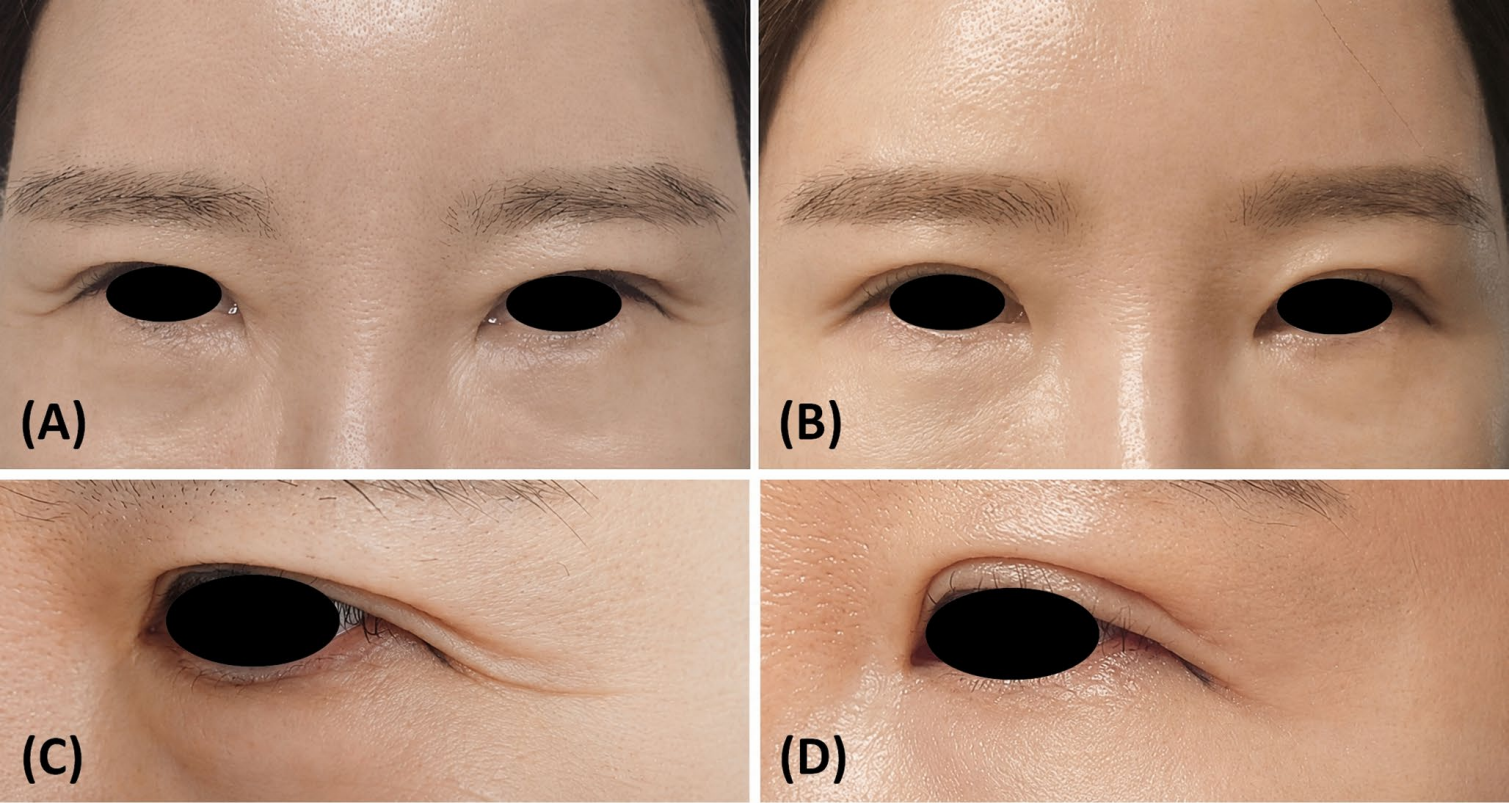
Clinical photographs of a 45-year-old female patient with Fitzpatrick skin type III. (**A**, **C**) Baseline (FWAS: 4). (**B**, **D**) Week 24 (FWAS: 3)

**Fig. 4:**
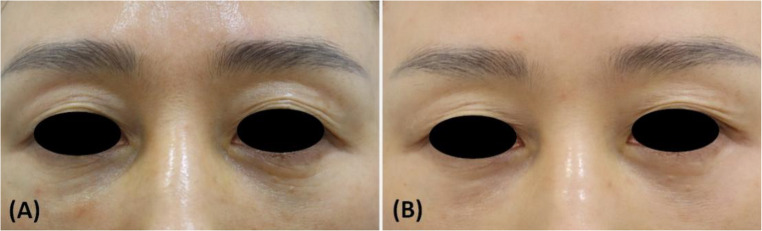
Clinical photographs of a 49-year-old female patient with Fitzpatrick skin type IV. (**A**) Baseline (FWAS: 6). (**B**) Week 24 (FWAS: 4)

**Fig. 5:**
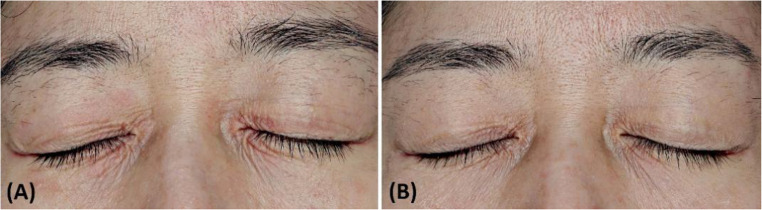
Clinical photographs of a 52-year-old female patient with Fitzpatrick skin type III. (**A**) Baseline (FWAS: 7). (**B**) Week 24 (FWAS: 5)

Representative clinical photographs of patients demonstrating a 2-point reduction in the Fitzpatrick Wrinkle Assessment Scale (FWAS) score at the 24-week follow-up compared to baseline are presented in Figs. 2, 3, 4 and 5.

The agreement between the two independent blinded evaluators for FWAS scoring was assessed using the Intraclass Correlation Coefficient (ICC). The reliability coefficient was 0.85 at baseline, 0.83 at week 16, and 0.88 at week 24. The overall reliability coefficient across all time points was 0.87, indicating excellent agreement.

### Three-dimensional imaging analysis

The mean average wrinkle depth was 0.22 ± 0.05 mm at baseline, 0.16 ± 0.04 mm at week 16, and 0.14 ± 0.03 mm at week 24. The corresponding values for maximum wrinkle depth were 0.65 ± 0.12 mm, 0.48 ± 0.09 mm, and 0.42 ± 0.08 mm, respectively. The indentation index was 29.85 ± 4.50 at baseline, 21.40 ± 3.80 at week 16, and 19.15 ± 3.20 at week 24. The overall temporal changes and post-hoc pairwise comparisons are summarized in Tables 3 and 4.

**Table 4:** Bonferroni-Corrected Post-hoc Comparisons

Outcome variable	Baseline vs. Week 16 *p*-value	Baseline vs. Week 24 *p*-value	Week 16 vs. Week 24 *p*-value
FWAS score	< 0.001	< 0.001	0.004
Average wrinkle depth	< 0.001	< 0.001	0.031
Maximum wrinkle depth	< 0.001	< 0.001	0.012
Indentation index	< 0.001	< 0.001	0.008
R2	< 0.001	< 0.001	< 0.001
R5	< 0.001	< 0.001	0.015
R7	< 0.001	< 0.001	0.022

*P*-values were adjusted using Bonferroni correction for multiple pairwise comparisons

### Skin elasticity

The mean R2 value was 0.55 ± 0.08 at baseline, 0.66 ± 0.06 at week 16, and 0.72 ± 0.05 at week 24. The mean R5 value was 0.35 ± 0.06, 0.44 ± 0.05, and 0.49 ± 0.04, respectively. The mean R7 value was 0.31 ± 0.05 at baseline, 0.38 ± 0.04 at week 16, and 0.43 ± 0.04 at week 24. Global longitudinal test results and Bonferroni-adjusted post-hoc comparisons are provided in Tables 3 and 4.

### Patient satisfaction

Patient satisfaction was assessed at week 16 and week 24 using a 5-point Likert scale. Satisfaction survey included scores in wrinkle reduction, elasticity improvement, overall periorbital appearance, and general procedural outcomes. The distribution of satisfaction scores is presented in Table 5. At week 16, the majority of patients (78.13%) reported being ‘satisfied’ or ‘very satisfied’ with the treatment, and this proportion increased to 90.63% at the final 24-week follow-up.

**Table 5:** Distribution of Patient Satisfaction Scores

Satisfaction score	Week 16, *n* (%)	Week 24, *n* (%)
1, very dissatisfied	0 (0.00%)	0 (0.00%)
2, dissatisfied	1 (3.13%)	0 (0.00%)
3, neutral	6 (18.75%)	3 (9.38%)
4, satisfied	16 (50.00%)	14 (43.75%)
5, very satisfied	9 (28.13%)	15 (46.88%)

### Pain and safety

The mean procedure time for a complete bilateral periorbital treatment session was notably brief at 7.2 ± 1.4 min. The overall mean pain score across all treatment sessions was 3.3 ± 0.9.

Safety was assessed immediately after each procedure and throughout the follow-up period. The most commonly observed post-treatment reactions were transient erythema and mild edema. Mild erythema was reported in all 32 patients (100.0%) and resolved within a mean duration of 1.25 ± 0.45 days. Mild edema occurred in 21 patients (65.6%) and resolved within 2.15 ± 0.85 days. All observed reactions required no medical intervention and resolved spontaneously without sequelae, resulting in a mean clinical downtime of 1.5 ± 0.5 days. No cases of bleeding, petechiae, bruising, crusting, thermal burns, persistent hyperpigmentation, infection, scarring, or sensory alterations were observed during the study period.

## Discussion

This study evaluated the 16- and 24-week clinical outcomes and safety of three sessions of microneedle RF treatment in adults with moderate-to-severe periorbital wrinkles. FWAS scores, Antera 3D wrinkle indices, Cutometer elasticity parameters, and patient satisfaction improved over time, while treatment-related reactions were limited to transient erythema and mild edema. These findings indicate that microneedle RF serves as a minimally invasive option for improving periorbital wrinkles and skin elasticity while offering short procedure times and limited recovery burdens.

Periorbital aging is among the earliest recognizable signs of facial aging. It involves complex structural changes beyond simple wrinkle formation. Periorbital skin is relatively thinner and possesses less subcutaneous fat and structural support compared to other facial areas. It remains constantly subjected to repetitive orbicularis oculi contractions, photoaging, and alterations in dermal collagen and elastic fibers [4, 11, 17]. These factors manifest as fine lines, static wrinkles, dynamic rhytides, skin laxity, and texture irregularities across the upper eyelid, lower eyelid, infraorbital region, and lateral canthus. Because lateral canthal lines and infraorbital wrinkles result from a combination of facial muscle contraction and dermal matrix degradation, superficial resurfacing or temporary muscle relaxation alone often fails to provide sufficient correction [4, 8].

Conventional periorbital rejuvenation methods are typically selected based on the primary clinical presentation. For prominent skin redundancy or eyelid ptosis, surgical approaches like blepharoplasty are effective. However, surgical interventions carry the risks of incisions, prolonged recovery, bruising, edema, scarring, and potential overcorrection or undercorrection. Botulinum toxin improves dynamic lateral canthal wrinkles associated with orbicularis oculi activity. However, its primary mechanism relies on muscle relaxation via chemodenervation. It does not directly induce dermal remodeling or skin elasticity improvements [8]. Laser resurfacing and chemical peels aim to remodel the epidermis and dermis, improving pigmentation and skin texture. Nevertheless, their application in the periorbital area requires caution due to the thin skin, propensity for hyperpigmentation, and extended recovery periods [2, 12]. Therefore, patients presenting with moderate to severe periorbital wrinkles and reduced elasticity who decline surgery or long recovery periods require a treatment strategy that selectively delivers energy to the dermis while minimizing epidermal damage.

Microneedle RF has evolved to bridge this therapeutic gap. Tissue electrical impedance converts RF energy into heat. Microneedles selectively deliver this thermal energy to preset depths within the dermis or superficial subdermal layer [5, 6]. Insulated needles offer a distinct advantage. They reduce unnecessary surface heat transfer along the needle shaft and create a relatively concentrated thermal coagulation zone at the distal needle tip [5, 10]. Previous studies have utilized microneedle fractional RF to improve periorbital wrinkles, facial fine lines, and skin laxity. Histologically, existing literature reports increases in dermal collagen, elastin, and fibrillin, along with changes associated with neocollagenesis and neoelastogenesis [6, 7, 14, 16]. These biological mechanisms support the reductions in wrinkle depth and enhancements in elasticity observed in the current study.

A key feature of the applied microneedle RF system is the integration of vacuum fixation, an insulated 25-pin tip, duty-controlled RF emission, and double-shot delivery within a single procedural step. The periorbital skin is thin and highly contoured. If the needle tip does not adhere stably, penetration depth and energy delivery can become uneven. Suction was utilized to stabilize the contact between the tip and the skin surface before needle insertion. This approach aimed to deliver RF energy more consistently to the preset depth, even in the highly mobile and irregular periorbital area. Additionally, the 25-pin insulated tip restricts surface heat transfer along the needle shaft. It is designed to generate selective thermal stimulation at the distal needle tip. This configuration helps concentrate thermal stimulation in the dermal target layer while reducing surface damage. This is particularly beneficial in areas like the eyelid and infraorbital region, where concerns regarding epidermal injury and pigmentary changes are high.

This protocol delivered sequential RF impulses to depths of 1.5 mm and 1.0 mm within a single needle insertion cycle. This approach distributes thermal stimulation to different target layers within the periorbital dermis. It simultaneously creates a deep dermal coagulation zone and a more superficial remodeling zone. Double-shot delivery offers the advantage of transferring RF energy to two distinct depths during the same puncture process, thereby improving procedural efficiency without increasing the number of needle passes. The completion of bilateral periorbital treatment sessions in an average of 7.2 ± 1.4 min suggests enhanced procedural efficiency in the restricted periorbital treatment area. Furthermore, duty control divides the RF emission into shorter sub-shots, which may reduce localized heat accumulation caused by continuous irradiation. However, given the observational study design, the specific contribution of dual-depth delivery and duty-controlled emission to the observed clinical outcomes cannot be independently determined. This depth-specific and duty-modulated energy delivery may have contributed to the observed improvements in FWAS scores, Antera 3D wrinkle indices, and Cutometer elasticity parameters. These findings suggest the combined effects of immediate collagen contraction and delayed dermal remodeling following RF thermal stimulation. RF energy induces immediate collagen fiber contraction. Subsequently, the wound-healing cascade triggers fibroblast activation, extracellular matrix deposition, and collagen and elastin remodeling [3, 6, 14]. A recent study comparing fractional microneedle RF and simple microneedling reported that microneedle RF alters the dermal fibroblast milieu and induces histological improvements related to collagen and elastin [7]. Although histological examinations were not included, the continuous improvements in FWAS, Antera 3D wrinkle depth, and Cutometer elasticity up to 24 weeks indicate that RF-induced tissue responses extend beyond short-term edema. These prolonged improvements support an association with sustained dermal remodeling over time.

Patient satisfaction further complements the clinical significance of the procedure. The proportion of patients reporting being “satisfied” or “very satisfied” increased from 78.13% at week 16 to 90.63% at week 24. Patients readily notice periorbital wrinkles in mirrors or photographs. This rise in satisfaction suggests that the wrinkle improvements observed in FWAS and 3D imaging translated into patient-perceived enhancements of periorbital appearance. Additionally, the procedure involved a brief mean treatment time of 7.2 ± 1.4 min, a mean pain score of 3.3 ± 0.9, and a limited mean clinical downtime of 1.5 ± 0.5 days. These limited procedural burdens likely contributed to high patient acceptability for periorbital rejuvenation. However, satisfaction evaluations remain subject to personal expectations, baseline severity, and individual recovery processes. Thus, interpreting satisfaction data alongside objective imaging and biomechanical measurements remains crucial.

The procedure demonstrated a stable safety profile. The most common post-treatment reactions were mild erythema and mild edema. Erythema occurred in all patients but resolved within a mean of 1.25 ± 0.45 days. Edema affected 21 patients (65.6%) and subsided within a mean of 2.15 ± 0.85 days. All reactions resolved spontaneously without medical intervention. No occurrences of bleeding, petechiae, bruising, crusting, thermal burns, persistent hyperpigmentation, infections, scarring, or sensory alterations were observed. The periorbital skin is thin, highly vascularized, and prone to visible pigmentary changes and edema, making these safety outcomes clinically relevant. The technological characteristics previously noted as primary mechanisms for tissue remodeling—stable adherence via vacuum fixation, prevention of surface heat accumulation via duty-controlled emission, and minimized mechanical trauma via the double-shot technique—appear to play a key role in reducing postoperative tissue damage alongside providing clinical efficacy. This confirms that the procedure can be performed safely on thin periorbital skin while maintaining a limited recovery burden.

Taken together, this modality does not aim to replace surgical interventions intended to correct severe eyelid ptosis or marked skin redundancy. Rather, it serves as an appropriate minimally invasive treatment option for patients presenting with moderate to severe periorbital wrinkles and diminished skin elasticity who decline surgical correction or extended recovery periods. The short procedure time, limited downtime, and absence of bleeding, pigmentation issues, or sensory alterations strongly support its clinical acceptability in periorbital rejuvenation, an area that frequently requires repeated treatments.

This study presents several limitations. The uncontrolled observational design makes it challenging to isolate RF-specific effects from physical needle stimulation or natural progression. Furthermore, the small, predominantly female cohort with Fitzpatrick skin types II to IV limits demographic generalizability. The 24-week follow-up is also insufficient to determine long-term outcome longevity or optimal retreatment intervals. Finally, without histological analysis, structural changes such as neocollagenesis remain indirectly inferred rather than directly verified. Future research should incorporate randomized controlled or split-face designs to validate independent efficacy. Additionally, investigating parameter dose-response relationships, evaluating responses across distinct periorbital anatomical subunits, and conducting extended follow-ups are necessary to optimize treatment protocols and confirm the cumulative safety of repeated procedures. In addition, despite standardized photography protocols, residual variability in lighting and exposure may still have influenced subjective wrinkle assessment and photographic comparisons.

## Data Availability

The data that support the findings of this study are available on request from the corresponding author. The data are not publicly available due to privacy or ethical restrictions.

## Declarations

### Clinical trial number

Clinical trial number: not applicable.
